# The Association Between Individualised Religiosity and Health Behaviour in Denmark: Are Social Networks a Mediating Factor?

**DOI:** 10.1007/s10943-022-01650-1

**Published:** 2022-09-09

**Authors:** Nanna Herning Svensson, Anders Larrabee Sonderlund, Sonja Wehberg, Niels Christian Hvidt, Jens Søndergaard, Trine Thilsing

**Affiliations:** Department of Public Health, Research Unit of General Practice, University of Southern Denmark, J. B. Winsløws Vej 9A, 5000 Odense, Denmark

**Keywords:** Religiosity, Social network, Diet, Physical activity, Mediation, Denmark

## Abstract

The present study investigates whether social networks mediate the well-established positive association between religiosity and health behaviour. Most research has focused on traditional public religiosity (e.g. regular church attendance). This study, however, focuses on the Danish population in which non-traditional and private religiosity is common. We utilise data from the Danish population-based project, *Early Detection and Prevention*. Our results suggest that religiosity is linked to health behaviour; however, this association is not mediated by social network.

**Supplementary Information:**

The online version contains supplementary material available at 10.1007/s10943-022-01650-1.

## Introduction

The positive association between religiosity and health is well-established in the literature (Koenig et al., 2012) and shows that religious individuals often tend to be healthier than their non-religious counterparts. Extensive empirical research has shown that this association may in part be due to better health behaviour (Koenig et al., 2012), with numerous studies showing links between religiosity and physical activity (Kobayashi et al., 2015), diet (Kim & Sobal, 2004; Reeves et al., 2012; Svensson et al., 2019), alcohol consumption (Koenig et al., 2012; Nordfjærn, 2018), and smoking (Kobayashi et al., 2015; Svensson et al., 2019). Recent studies have also shown that religiosity is associated with a later sexual debut (Moreau et al., 2013) and safe sex (Vigliotti et al., 2020). Nevertheless, exactly how and why religiosity facilitates health behaviour is unclear.

Several studies have argued that the positive association between religiosity and health behaviour may be mediated by the social aspects that often are associated with religiosity. That is, religion, like most other forms of group memberships, may provide a sense of social identity, norms, belonging, and community for its adherents (Ellison & Levin, 1998). This type of social connectedness represents a source of emotional and practical social support which likely confers a broad range of health benefits on the individual (Haslam et al., 2008; Haslam et al., 2009; Lim & Putnam, 2010; Ysseldyk et al., 2013). For example, both Ellison and Levin (1998) and Oman and Thoresen (2002) have proposed that the social networks and support systems that are accessible through religious participation represent one potential pathway through which religiosity impacts on health behaviour. They argue that the positive health values and norms attached to many religious groups and communities facilitate individual health behaviour and discourage unhealthy behaviour. They also note the potential positive effects of religious coping methods that buffer against psychological stress, many of which are rooted in community cohesion and social support (Ellison & Levin, 1998; Oman & Thoresen, 2002). Other research has found that being embedded in a supportive and religious social network provides health-oriented social capital in the form of health resources and information as well as moral and practical support to engage in health behaviour (Yeary et al., 2012). Additional studies have demonstrated that the social support received from a church congregation predicted moderately increased levels of physical activity, greater fruit and vegetable consumption, and less tobacco use compared to a general (i.e. non-religiosity specific) social support measure (Debnam et al., 2012). These studies thus indicate that the positive link between religiosity and health behaviour may be mediated by religious social networks and the associated support.

While the association between religiosity, social network, and health behaviour is relatively clear, the aforementioned studies have exclusively focused on support derived from *traditional* participatory, public religiosity such as being an active member of a church congregation. The reason for this presumably relates to the fact that most of the research in this area has come out of the US, arguably the most fervently religious nation in the West where a particularly public, participatory, and socially oriented brand of Christianity permeates most parts of society ("Americans are far more religious than adults in other wealthy nations," 2018; "U.S. adults are more religious than Western Europeans," 2018). However, outside of (as well as within) the US, there are other styles of religiosity that rely less on explicit social participation in well-defined and highly visible faith-based organisations, but which still may facilitate increased and more diverse social connectedness through other mechanisms. For example, several studies have found that while traditional, public religious social networks may provide ready access to a cohesive, supportive, and distinctive in-group (typically defined by denomination, congregation, etc.), the often exclusive nature of this network might also prevent potentially valuable outgroup social relationships (e.g. with secular individuals or people of other religious convictions), thus limiting the diversity of the individual’s social network (Cheadle & Schwadel, 2012). By contrast, private religiosity—characterised by quiet faith and less explicit religious activity and participation—has been linked with less restrictive social interaction and more expansive social values, universalism, and openness to people regardless of creed (Schwadel & Hardy, 2022). In other words, while *public religiosity* may provide membership in a clearly defined but often relatively rigid and exclusive in-group, *private religiosity* may facilitate more diverse, flexible, and less prescriptive social connectedness. Consistent with this, Hastings (2016) found that people who were spiritual but non-denominational were no less connected than people who engaged in denominational and public religious activity (e.g. regularly attending church services) (Hastings, 2016). Thus, in terms of social connectedness, there was no significant advantage associated with traditional public religiosity over non-denominational and private faith or spirituality. This suggests a generalised social element of religious faith that exists in addition to the distinct and potentially exclusive in-group communities that are associated with traditional public denominational religiosity. These findings beg the question of whether private, non-traditional religiosity unlocks the same health benefits via social connectedness as traditional public denominational religious participation often does.

To our knowledge, no studies have been conducted on the link between private religiosity, social connectedness, and health behaviour (Debnam et al., 2012; Yeary et al., 2012). In the bid to fill this gap in the literature, the Danish population is particularly interesting and relevant. Non-traditional and private religiosity is common in Denmark, often involving individual rather than communal worship and typically representing a more generalised and loosely defined spirituality and faith (Andersen & Lüchau, 2011). The Danish population may therefore be especially well-suited for studying the impact of public versus private faith on social connectedness and any associated health behaviours. Indeed, previous research has found that religiosity in a Danish population was associated with several health benefits, including healthier diet and less smoking (Svensson et al., 2019). While this study did not tap social connectedness as a potential mediator of this link, given the research discussed in the previous paragraph, this may nonetheless have been a likely mechanism.

In this paper, we focus on whether the Danish brand of quiet and solitudinous religiosity facilitates social connection and health behaviour to the same extent as more traditional social and participatory religiosity. To explore this question, in the present article, we distinguish between two forms of religiosity: (1) *public religiosity* and (2) *private religiosity*. The former is defined by the individual’s centralised denominational social identification. This may manifest in terms of their active and open engagement with typical religious social behaviours and group memberships, including regular church/mosque/temple attendance, group prayer (e.g. before a meal), congregation-driven community initiatives (e.g. charities, fundraisers), and social activities, etc. This is the type of religiosity most commonly studied in the empirical literature. Private religiosity, on the other hand, indicates a more reserved, moderate, and less-participatory style of religiosity (e.g. praying alone, rarely participating in religious events and practices). Here, the individual may still identify as religious or spiritual, but their social interactions may not be dominated by this particular aspect of their self-concept to the same degree as their more traditional and publicly religious counterparts. Rather than limit their social connectedness, this may facilitate a more diverse and less exclusive range of available social interactions and networks.

### Hypotheses

We theorise that in spite of the perceived less social nature of private religiosity, this religious style may actually encourage social connectedness to a similar extent as traditional public religiosity and with similar implications for health behaviour (Ellison & Levin, 1998). In other words, the mechanisms that underpin the known link between religiosity and health behaviour may not pertain exclusively to *religious* social networks (derived through public religiosity), but may also relate to social connectedness that stems from a more general openness to others (characteristic of private religiosity). Specifically, we hypothesise that (1) religious individuals have healthier lifestyles compared to individuals with no religiosity, (2) religiosity is positively associated with social network strength, and (3) the strength of the individual’s social network mediates the association between religiosity and lifestyle.

## Methods

### Setting and Study Population

We used pre-collected data from the population-based TOF pilot2 study (TOF is the Danish acronym for *Early Detection and Prevention*)—a pilot study testing the feasibility and acceptability of a targeted intervention to promote healthy lifestyle and reduce the risk of lifestyle-related diseases. The intervention comprised an initial invitation, a subsequent risk stratification based on self-reported lifestyle and electronic patient record data, and finally a targeted intervention specifically for patients with health-risk behaviour or high risk of disease ("An Adjusted Preventive Program Against Lifestyle Related Diseases (TOFpilot2)," 2019). TOF pilot2 was implemented in two Danish municipalities (Middelfart and Haderslev) in 2018–2019. The initial invitation was sent to all 29- to 60-year-old patients affiliated with 15 participating general practitioners (GPs) (*n* = 6347) (Thilsing et al., 2021). The vast majority of the Danish population (> 99%) is affiliated with a GP ("Stort fald i antallet af gruppe 2-patienter," 2017). Patients were excluded if they did not reside in either of the two municipalities, had name or address protection, participated in the first TOF pilot study (TOF pilot1 conducted in 2016–2017 (Larsen et al., 2018)), or did not have a digital mailbox (e-Boks) to which the invitation could be sent. E-Boks is a secure platform for digital communication between public authorities and other trusted organisations. Over 95% of the population in Denmark have an e-Boks account ("Statistik om Digital Post," 2020). Patients who consented to participate were asked to fill in an online questionnaire (Q1) that included questions on social network and religiosity. Approximately three months later, all participants were asked to fill in another online questionnaire (Q2) concerning health-status, health behaviours, and family history of specific diseases. Q1 consisted of 42 categorical questions with no forced response options, whereas Q2 consisted of 39 questions with forced response options only. No targeted intervention was offered until after Q2.

### Outcomes

The Q2 questionnaire included information on self-reported diet and level of physical activity. Diet was measured based on consumption of fish, fruit, vegetables, and sweets. A total dietary score was calculated on the basis of these items, ranging from 0 to 12. The variable was dichotomised into healthy (total score of five or above) or unhealthy (score below five) categories (Socialstyrelsen, 2011). Physical activity was measured with one item, gauging the level of physical activity in the past 12 months. Response categories included “*Training hard and participating in competitive sports regularly and several times a week*”, “*Participate in recreational sports or perform heavy gardening or similar activities at least 4 h a week*”, “*Walk, cycle or do other light exercise at least 4 h a week*” and “*Read, watch television, or other sedentary activities*” (Christensen et al., 2004). The variable was dichotomised to reflect a sedentary lifestyle (the last of the mentioned response options) versus an active one (the first three response options). This measure is based on The Danish Health Authority’s recommendations for physical activity for adults aged 18–64 years ("Anbefalinger om fysisk aktivitet for voksne under 65 år," 2019). There were no missing values as the questionnaire used forced response options.

### Independent Variables; Prayer/Meditation Practice and Church/Mosque Attendance

Information about prayer/meditation practice and church/mosque attendance was obtained from Q1 and based on the wording of the questions from The Danish Value Survey 2008, a part of The European Values Survey ("Den danske værdiundersøgelse 1981–2017," 2019). The response categories for prayer/meditation practice were adapted to match the categories from The Sources of Meaning and Meaning in Life Questionnaire (SoMe) (Schnell, 2009). The response categorisation for church/mosque attendance originated from The Danish Values Survey ("Den danske værdiundersøgelse 1981–2017," 2019). Some of the original response categories were merged as listed below.

**Fig. 1:**
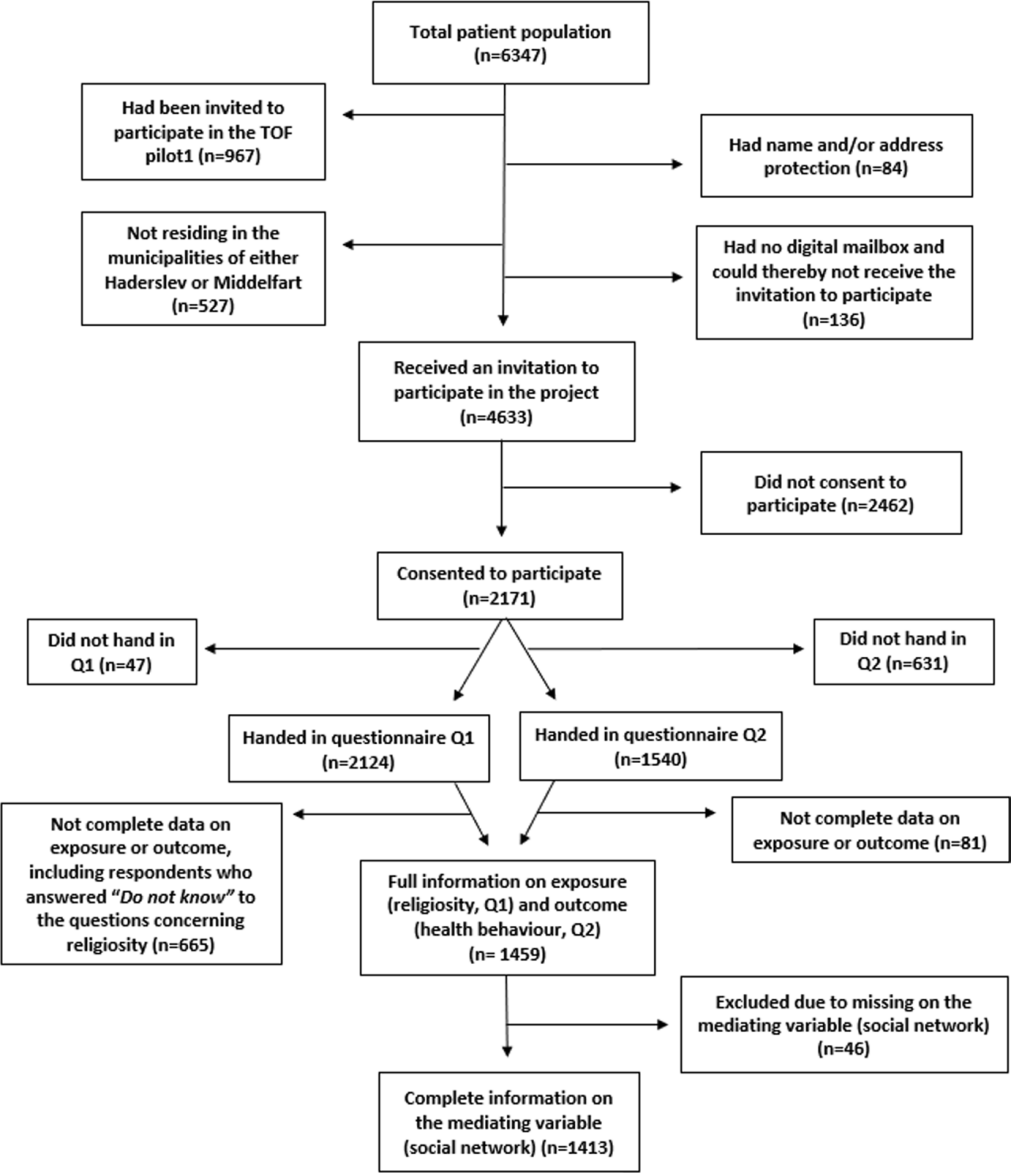
Flowchart over patient population

Prayer/meditation was assessed by asking respondents about the extent to which they pray/meditate, “*It happens that I pray, meditate, or the like”* (1: “*To a very great extent*”, 2: “*To a great extent*”, 3: “*To some extent*”, 4: “*Not at all*” and 5: “*Do not know*”). Church/mosque attendance was assessed by asking respondents how often they attended religious events, “*How often do you go to church, mosque or another religious community event? Please do not include weddings, funerals, and christenings”* (1: “*At least once a month*”, 2: “*Less than once a month*”, 3: “*On special occasions (like Christmas and Easter)*”, 4: “*Never, almost never*” and 5: “*Do not know”*)*.* The variables were dichotomised such that response categories 1, 2, and 3 represented the category *open towards either prayer/meditation practice or church/mosque attendance* and response category 4 (“*Never, almost never*”) represented the category *closed towards either prayer/meditation practice or church/mosque attendance.* If participants responded, “*Do not know”* (category 5), their entire response was excluded from the statistical analysis as this answer contained no information about religiosity (Fig. 1). We created one combined exposure variable and categorised it as 0: No religiosity (0P0C)—closed towards both prayer/meditation practice and church/mosque attendance, 1: Public religiosity-church only (0P1C)—closed towards prayer/meditation practice but open towards church/mosque attendance, 2: Private religiosity-prayer only (1P0C)—open towards prayer/meditation practice but closed towards church/mosque attendance, and 3: Public religiosity-church and prayer 1P1C—open towards both prayer/meditation practice and church/mosque attendance. Thus, the four categories—that are based on items that tap religious behaviour—act as proxies for different degrees of religiosity. Group 0P0C represents people with no religiosity, groups 0P1C and 1P1C represent public religiosity, and group 1P0C represents private religiosity.

### Mediating Factor: The Lubben Social Network Scale (Social Network Strength)

The Lubben Social Network Scale (LSNS-6) consists of six items. Three concern relatives and three concern friends: “*How many relatives do you see or hear from at least once a month?*”, “*How many relatives do you feel at ease with that you can talk about private matters?*”, and “*How many relatives do you feel close to such that you could call them for help?”*. Response categories were 0: “*None*”, 1: “*One*”, 2: “*Two*”, 3: “*Three or four*”, 4: “*Five through eight*” and 5: “*Nine or more*” (Lubben et al., 2006). The three questions were repeated with the word *friends* instead of *relatives*. The LSNS-6 thus measures both the extent (in terms of quantity of the individual’s social ties) and quality (in terms of the social support derived from these social ties) of the individual’s social network. The total score for the LSNS-6 ranges from 0 to 30 with a recommended cut point of 12, where respondents with a total score of less than 12 are categorised as having a weak social network (Lubben et al., 2006). Respondents who did not answer all six items were excluded (*n* = 46) (see Fig. 1). For our analyses, we created both a continuous sum score and a binary variable (strong or weak social network with a cut point of 12), based on the number and the quality of individual social ties.

Covariates included sex [“Men” and “Women”], age group [“29–39 years”, “40–49 years” and “50–60 years”], level of education [“ ≤ 10 years”, “10–15 years” and “ > 15 years”], cohabitation status [“Cohabiting” and “Single”], country of origin [“Denmark” and “Not Denmark”], and employment status [“Employee/selfemployed” and “Not employed”]. The option “Not employed” covered recipients of social security pay outs, retirement benefits, and state education grants. Information on sex, age, level of education, cohabitation status, country of origin, and employment status was retrieved from the national Danish Bureau of Statistics (Statistics Denmark).

## Statistical Analysis

**Fig. 2:**
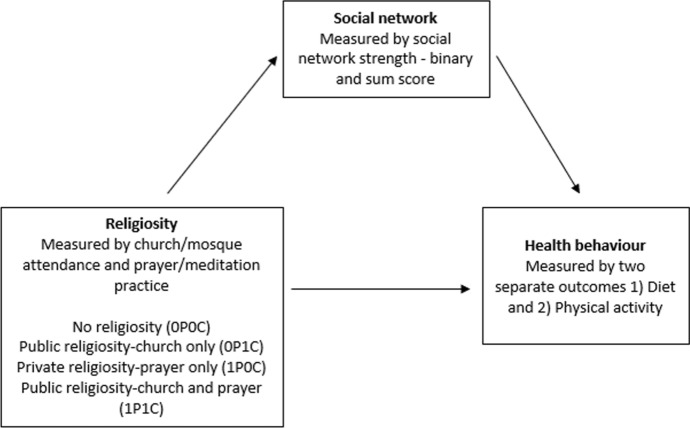
Construction of the unadjusted mediation model—In the adjusted models, the following variables were included: sex, age group, level of education, cohabitation status, country of origin, and employment status

Descriptive statistics were reported according to respondent religiosity category (open or closed towards prayer/meditation practice and/or church/mosque attendance), using the following terms no religiosity (0P0C), public religiosity-church only (0P1C), private religiosity-prayer only (1P0C), and public religiosity-church and prayer (1P1C) (the definition is outlined in section *Independent variables; prayer/meditation practice and church/mosque attendance* and can also be found under the descriptive table). Two separate outcomes were used, namely *healthy diet* and *physical activity.* All analyses were conducted with both the binary social network variable (referred to as model 1) and the continuous social network sum score variable (referred to as model 2) as a potential mediator. For all associations of interest both unadjusted and adjusted analyses were performed. The overall model is depicted in Fig. 2. The unadjusted and adjusted mediation analyses were performed in *R*, with the *R* package medflex. The weighting-based approach was used to estimate the *natural direct effect* (NDE)*, natural indirect effect* (NIE), and *total effect* (TE), all presented as odds ratios with a corresponding 95% confidence interval (CI). Likewise, logistic regression models were also applied estimating odds ratios. We applied a statistical significance cut-off level of 5% to all analyses. Logistic regression analyses were performed in Stata version 16.1 (StataCorp LLC).

## Results

### Descriptive Statistics

Of the total patient population (*N* = 6347), 4633 individuals were invited to participate and 2171 consented to take part in the study. Of these, a total of 2124 filled in the Q1 questionnaire and 1540 filled in the Q2 questionnaire. Ultimately, 1413 responses had no missing data on exposure, outcome, or the potentially mediating variable and were therefore included in the analysis (Fig. 1). In general, response rates were highest among patients between 50–60 years of age, with a medium-long (10–15 years) to long (> 15 years) education, and who were cohabiting with a partner (Table 1). Approximately one third (*n* = 453) of the respondents were categorised as ‘no religiosity’ (0P0C—no prayer, no church), closely followed by respondents in the category ‘public religiosity-church and prayer’ (*n* = 446) (1P1C—yes prayer, yes church). Women were more likely than men to be in the categories ‘public religiosity-church and prayer’ (1P1C, women: 74.0% vs. men: 26.0%), ‘public religiosity-church only’ (0P1C, women: 60.0% vs. men: 40.0%), and ‘private religiosity-prayer only’ (1P0C, women: 74.0% vs. men: 26.0%), while men were more likely than women to be in the category ‘no religiosity’ (0P0C, men: 58.5% vs. women: 41.5%).

**Table 1:** Characteristics of the respondents by prayer/meditation practice and church/mosque attendance

		No religiosity (0P0C)	Public religiosity-church only (0P1C)	Private religiosity-prayer only (1P0C)	Public religiosity-church and prayer (1P1C)	Total
	Total [*n* (%)]	453 (100.0)	295 (100.0)	219 (100.0)	446 (100.0)	1413 (100.0)
Social network	Strong (score ≥ 12)	385 (85.0)	274 (92.9)	192 (87.7)	419 (93.9)	1270 (89.9)
	Weak (score < 12)	68 (15.0)	21 (7.1)	27 (12.3)	27 (6.1)	143 (10.1)
Sex	Men	265 (58.5)	118 (40.0)	57 (26.0)	116 (26.0)	556 (39.3)
	Women	188 (41.5)	177 (60.0)	162 (74.0)	330 (74.0)	857 (60.7)
Age group	29–30 years	83 (18.3)	52 (17.6)	45 (20.5)	71 (15.9)	251 (17.8)
	40–49 years	160 (35.3)	112 (38.0)	75 (34.2)	195 (43.7)	542 (38.4)
	50–60 years	210 (46.4)	131 (44.4)	99 (45.2)	180 (40.4)	620 (43.9)
Country of origin	Denmark	439 (96.9)	291 (98.6)	211 (96.3)	430 (96.4)	1371 (97.0)**
	Not Denmark	14 (3.1)	4 (1.4)	8 (3.7)	16 (3.6)	42 (3.0)
Level of education	≤ 10 years	87 (19.2)	34 (11.5)	35 (16.0)	48 (10.8)	204 (14.4)
	10–15 years	232 (51.2)	144 (48.8)	107 (48.9)	200 (44.8)	683 (48.3)**
	> 15 years	134 (29.6)	117 (39.7)	77 (35.2)	198 (44.4)	526 (37.2)
Cohabitation status	Cohabiting	351 (77.5)	241 (81.7)	160 (73.1)	358 (80.3)	1110 (78.6)**
	Single	102 (22.5)	54 (18.3)	59 (26.9)	88 (19.7)	303 (21.4)
Employment status	Employee/self-employed	389 (85.9)	271 (91.9)	180 (82.2)	392 (87.9)	1232 (87.2)
	Not employed*	64 (14.1)	24 (8.1)	39 (17.8)	54 (12.1)	181 (12.8)

^*^Includes among others, receivers of social security pay outs, retirement benefits and state education grants

^**^Missing have been included under the majority (country of origin and cohabitation status* n* =  <  5, and level of education* n* = 16)

^***^Note: No religiosity (0P0C): Closed towards both prayer/meditation practice and church/mosque attendance, public religiosity-church only (0P1C): Closed towards prayer/meditation practice but open towards church/mosque attendance, private religiosity-prayer only (1P0C): Open towards prayer/meditation practice bud closed towards church/mosque attendance, and public religiosity-church and prayer (1P1C): Open towards both prayer/meditation practice and church/mosque attendance

**Fig. 3:**
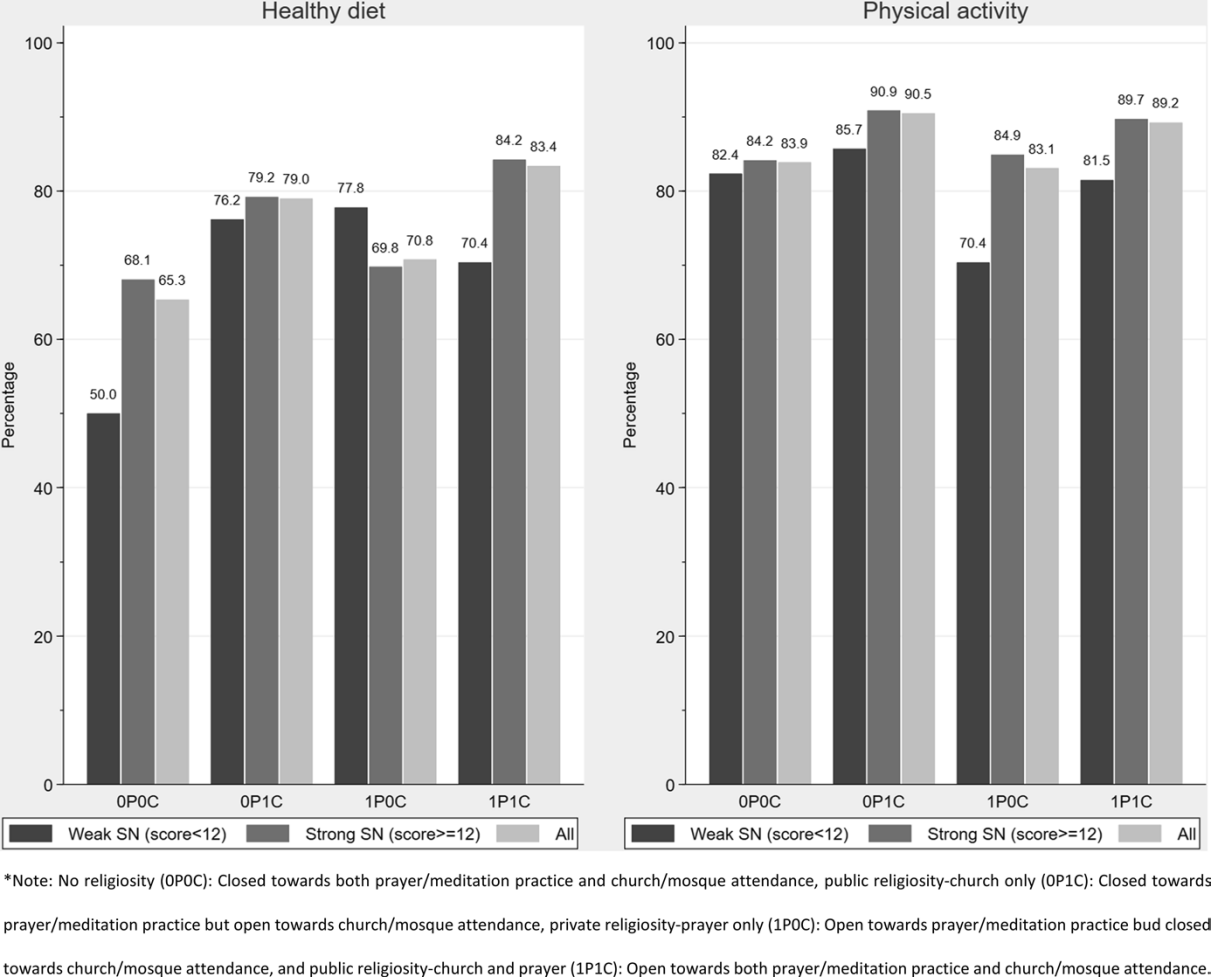
Diet and level of physical activity across the four religion categories by strength of social network

Across all levels of religiosity, the vast majority of respondents reported being physically active, ranging from 83.1 to 90.5%. Similarly, most respondents reported having a healthy diet, ranging from 65.3 to 83.4% (Fig. 3).

Only 10.1% (*n* = 143) of the study sample had weak social networks. Just under half of these (47.5%, *n* = 68) reported no religiosity (0P0C) with the rest being fairly evenly distributed across the other three categories (public religiosity-church only (0P1C) = 14.7%, *n* = 21—private religiosity-prayer only (1P0C) = 18.9%, *n* = 27—public religiosity-church and prayer (1P1C) = 18.9%, *n* = 27). Thus, among people with weak social networks, the category ‘no religiosity’ (0P0C) was 2.5 to 3 times as large as the categories ‘public religiosity-church only’ (0P1C), ‘private religiosity-prayer only’ (1P0C), and ‘public religiosity-church and prayer’ (1P1C). By contrast, among respondents with strong social networks there was no clear pattern. Most people with strong networks were either in the ‘public religiosity-church and prayer’ (1P1C, 33%, *n* = 419) or ‘no religiosity’ (0P0C, 30.3%, *n* = 385) categories, followed by people in the ‘public religiosity-church only’ (0P1C), 21.6%, *n* = 274 and ‘private religiosity-prayer only’ (1P0C), 15.1%, *n* = 192) categories (Supplementary Table 1). However, looking at the continuous social network sum score variable, there were no significant differences between the four categories. People in the ‘public religiosity-church and prayer’ category (1P1C) had the highest mean social network sum score (sum score = 20), closely followed by ‘public religiosity-church only’ (0P1C) with a mean social network sum score of 19. The categories ‘no religiosity’ (0P0C) and ‘private religiosity-prayer only’ (1P0C) both had a mean social network sum score of 18 (Supplementary Fig. 1).

Figure 3 depicts the interaction between religiosity, social network strength, and health behaviour. In terms of proportion of respondents who reported having a healthy diet, a general (light grey bars) upwards trend is apparent from no religiosity (0P0C, 65.3%, *n* = 295) to public religiosity-church and prayer (1P1C, 83.4%, *n* = 372). This increasing trend is nearly identical for people with strong social networks (grey bars), of whom 89.9% had strong social network. Thus, for people with strong social networks, there was a positive association between healthy diet and religiosity. A similar, albeit weaker, association was evident among people with weak social networks. Despite the higher percentage of people who were physically active, there was no obvious pattern of interaction. The religiosity categories were fairly comparable in terms of social network strength and physical activity, barring a relative dip in physical activity for people in the category private religiosity-prayer only (1P0C) who had weak social networks. The highest percentage of individuals who reported being physically active and who had strong social networks were in the category ‘public religiosity-church only’ (0P1C, 90.9%, *n* = 268) (Fig. 3).

### The Mediating Effect of Social Network in the Association Between Religiosity and Health Behaviour

Results from the medflex analysis for both model 1 (social network operationalised as a binary variable) and model 2 (social network as a continuous sum score variable) show no significant mediating effect (NIE = Natural indirect effect) of social networks in the association between religiosity and healthy diet for any of three categories of religiosity. The indirect effect was either non-significant or very close to one (public religiosity-church only (0P1C), OR: 1.07, 95% CI: 1.02–1.14 and public religiosity-church and prayer (1P1C), OR: 1.09, 95% CI: 1.02–1.16). Therefore, the direct effect (NDE = Natural direct effect) was close to the total effect (TE = Total effect), and the results on the total effect from the medflex analysis were similar to results from the logistic regression analysis (Table 2 and supplementary Table 2).

**Table 2:** Results from the medflex analyses

		Healthy diet				Physical activity			
		Model 1		Model 2		Model 1		Model 2	
		Unadjusted OR (95% CI)	Adjusted OR (95% CI)	Unadjusted OR (95% CI)	Adjusted OR (95% CI)	Unadjusted OR (95% CI)	Adjusted OR (95% CI)	Unadjusted OR (95% CI)	Adjusted OR (95% CI)
No religiosity (0P0C)		ref	ref	ref	ref	ref	ref	ref	ref
Public religiosity-church only (0P1C)	NDE	1.91 (1.37;2.67)	1.66 (1.15;2.37)	1.76 (1.25;2.49)	1.55 (1.07;2.22)	1.79 1.09;2.885)	1.67 (1.00;2.63)	1.59 (0.97;2.51)	1.53 (0.92;2.43)
	NIE	1.04 (1.00;1.09)	1.02 (0.99;1.06)	1.11 (1.04;1.18)	1.07 (1.02;1.14)	1.05 (0.99;1.11)	1.03 (0.98;1.08)	1.16 (1.06;1.26)	1.11 (1.03;1.20)
	TE	1.99 (1.43;2.78)	1.70 (1.18;2.42)	1.95 (1.37;2.77)	1.67 (1.15;2.38)	1.87 (1.14;2.98)	1.72 (1.03;2.71)	1.83 (1.12;2.89)	1.70 (1.02;2.71)
Private religiosity-prayer only (1P0C)	NDE	1.22 (0.87;1.73)	1.03 (0.71;1.49)	1.18 (0.82;1.69)	0.99 (0.68;1.43)	0.96 (0.61;1.50)	0.91 (0.57;1.45)	0.90 (0.58;1.39)	0.88 (0.53;1.40)
	NIE	1.01 (0.98;1.05)	1.01 (0.98;1.04)	1.03 (0.98;1.08)	1.03 (0.99;1.07)	1.02 (0.98;1.06)	1.02 (0.97;1.05)	1.04 (0.98;1.11)	1.04 (0.98;1.11)
	TE	1.24 (0.88;1.76)	1.04 (0.72;1.50)	1.22 (0.85;1.75)	1.02 (0.70;1.48)	0.98 (0.61;1.53)	0.93 (0.58;1.47)	0.94 (0.60;1.45)	0.91 (0.55;1.47)
Public religiosity-church and prayer (1P1C)	NDE	2.46 (1.80;3.39)	2.02 (1.41;2.86)	2.32 (1.68;3.19)	1.90 (1.33;2.71)	1.53 (1.00;2.29)	1.45 (0.94;2.20)	1.36 (0.89;2.07)	1.33 (0.86;1.97)
	NIE	1.05 (1.00;1.10)	1.03 (0.99;1.07)	1.12 (1.05;1.19)	1.09 (1.02;1.16)	1.05 (0.99;1.12)	1.04 (0.98;1.10)	1.17 (1.08;1.28)	1.14 (1.05;1.23)
	TE	2.58 (1.90;3.53)	2.08 (1.46;2.94)	2.60 (1.89;3.57)	2.08 (1.45;2.96)	1.61 (1.06;2.40)	1.51 (0.98;2.27)	1.59 (1.05;2.41)	1.51 (0.97;2.25)

^*^Model 1 refers to the model including the binary social network variable whereas model 2 refers to the model including the continuous social network sum score variable

^**^Adjusted for sex, age group, level of education, cohabitation status, country of origin, and employment status

^***^NDE = Natural direct effect, NIE = Natural indirect effect, and TE = Total effect. NIE shows the effect of the independent variable that goes through the mediator

^****^Note: No religiosity (0P0C): Closed towards both prayer/meditation practice and church/mosque attendance, public religiosity-church only (0P1C): Closed towards prayer/meditation practice but open towards church/mosque attendance, private religiosity-prayer only (1P0C): Open towards prayer/meditation practice bud closed towards church/mosque attendance, and public religiosity-church and prayer (1P1C): Open towards both prayer/meditation practice and church/mosque attendance

**Fig. 4:**
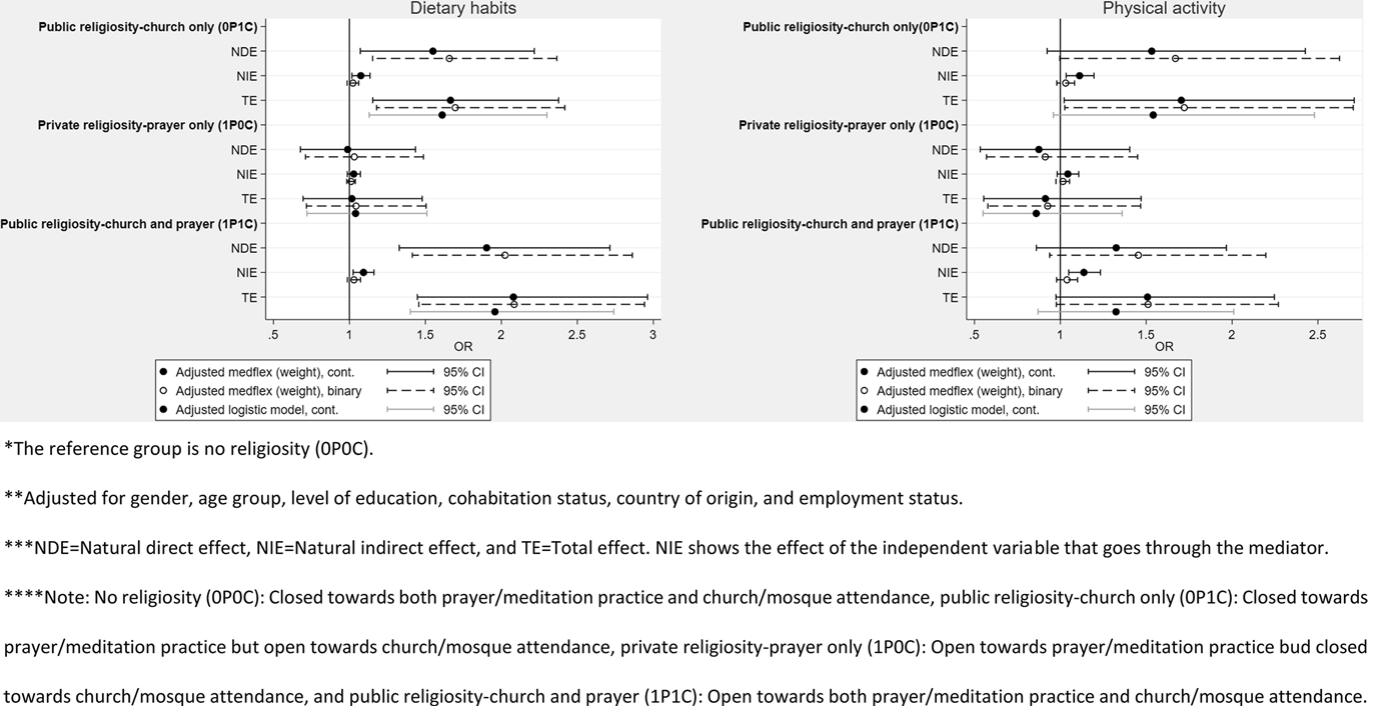
Results from the adjusted medflex and logistic regression analysis on religiosity related to diet and physical activity and social network strength

We detected no difference in terms of healthy diet between the reference group (no religiosity) and the group categorised as private religiosity-prayer only (1P0C) neither in model 1 or model 2. Conversely, results from model 1 showed that the public religiosity-church only (0P1C, OR: 1.66, 95% CI: 1.15–2.37) and public religiosity-church and prayer (1P1C, OR: 2.02, 95% CI: 1.41–2.86) categories were significantly more likely to have a healthy diet than the no religiosity (0P0C) category. Like model 1, the analysis for model 2, showed statistically significant *direct* results indicating that participants categorised as ‘public religiosity-church only’ (0P1C, OR: 1.55, 95% CI: 1.07–2.22) and ‘public religiosity-church and prayer’ (1P1C, OR: 1.90, 95% CI: 1.33–2.71) had significantly healthier diet compared to participants categorised as ‘no religiosity’ (0P0C) (Fig. 4 and Table 2).

As with healthy diet, no significant mediating effect of social networks was detected in the association between religiosity and physical activity in either model 1 or model 2. Mediation results in both models were either close to one (model 2: public religiosity-church only (0P1C), OR: 1.11, 95% CI: 1.03–1.20 and public religiosity-church and prayer (1P1C), OR: 1.14, 95% CI: 1.05–1.23) or non-significant. Therefore, the direct effects were close to the total effects. The results from simple logistic regression models were comparable (Table 2 and supplementary Table 2).

In both model 1 and model 2, no statistically significant direct effect was detected. Indicating no difference between the four categories of religiosity (public religiosity-church only (0P1C), private religiosity-prayer only (1P0C), and public religiosity-church and prayer (1P1C)) and physical activity compared to the reference group (no religiosity (0P0C)) (Fig. 4 and Table 2).

Overall, the results for the mediating effect of social network in the association between religiosity and both healthy diet and physical activity revealed comparable patterns, albeit with slightly less-pronounced effects for physical activity.

## Discussion

This study investigated the extent to which the strength (in terms of extent and quality) of people’s social networks mediates the association between religiosity (public and private) and health behaviour (healthy diet and physical activity). Our results support our first hypothesis that religious individuals have healthier lifestyles compared to individuals with no religiosity. Specifically, we found that people who were publicly religious (0P1C & 1P1C) were more likely to have a healthy diet than people with no religiosity (0P0C). We did not find support for our second hypothesis that religiosity (public or private) is positively associated with social network strength. Nor did we find any evidence that social network strength mediated the association between religiosity and health behaviour (hypothesis 3). Finally, we found that the relationship between religiosity and diet differed between private and public religious groups. That is, those who were categorised as ‘public religious’ (0P1C and 1P1C) were more likely to have a healthy diet than those categorised as ‘no religiosity’ (0P0C), whereas no such difference was seen between ‘private religiosity-prayer only’ (1P0C) and ‘no religiosity’ individuals. Thus, our results indicate that public religiosity (as opposed to private and no religiosity) is positively linked with a healthy lifestyle—especially in terms of diet. This effect, however, does not seem to be mediated by social network strength.

Our results align with past research. A previous study found a positive association between public religiosity (measured by church and/or mosque attendance) and healthy lifestyle. Similar to the present research, the study was based on a sample of Danes (Svensson et al., 2019). Results from previous research investigating whether social support and/or social networks mediate the association between religiosity and different aspects of physical and mental health show inconsistent results (Holt et al., 2018, 2013; Kim & Sobal, 2004).

A possible reason that no social network mediation was detected in the present study might be to do with the fact that there was very little variance in the social network sum score between the four categories of religiosity (Supplementary Fig. 1).

### Strengths and Limitations

The present study has both strengths and limitations. This study uses a cross-sectional design, preventing any conclusions about causality. Nonetheless, the mediation analyses in this study assume directional causality. These assumptions are regularly applied in logistic regression models, even though we do not know the true nature of the direction and therefore, cannot rule out the possibility of reverse causation. Another limitation worth mentioning is the lack of knowledge about the timewise sequence of religiosity. Depending on why an individual becomes religious might affect health behaviour. For example, is deeply-rooted and longstanding faith (restful religiosity) more likely to be associated with health as opposed to short-term “crisis” religiosity (such as that triggered by, for instance, illness or grief) (Hvidt et al. 2017).

Another limitation is the possible role of information bias and selection bias, which often occurs using self-administered questionnaires. The basic characteristics of the study population revealed that respondents were more likely to be women, cohabitating, and with higher socio-economic status, and tended to have a healthy diet, be physically active, and have strong social networks. In other words, due to the lack of variance in data, we cannot rule out that the results of the present study would be different if the study population had been less homogenous. Furthermore, the possible effect of recall bias cannot be excluded, especially in relation to the question concerning level of physical activity which asks participants to recall their level of activity during the past 12 months.

The variables used to measure religiosity in the study represent strengths and limitations as well. Prayer and church attendance are two widely applied measures within this field of research (Hall et al. 2008; Koenig et al., 2012). Nonetheless, it is very challenging to measure something as complex as religiosity (Hall et al., 2008). For instance, using prayer or meditation as a proxy for religiosity has been found to correlate negatively with depressive symptoms (Ahrenfeldt et al., 2017). These inconsistencies may reflect the variability in whether the individual prays or meditates as part of a regular routine or only in the face of crisis, or whether meditation is religious at all. Further, people might attend church or mosque for other reasons than religiosity, such as a choir recital and studies. When comparing results from other studies, it is important to note that Scandinavians in general report lower rates of religiosity compared to other populations and in particular Americans—one of the most studied nationalities in terms of the religiosity-health connection ("5 facts about the religious lives of African Americans," 2018; Zuckerman, 2008).

In terms of whether this study population identifies as religious or not, has not been included in the analysis—we have used religious activities and behaviours, such as prayer/meditation and church/mosque attendance, as proxies for religious identification and faith. Furthermore, Danes in general tend to describe themselves as believers but not religious (Rosen, 2009). This presents a problem when distinguishing—or whether to distinguish at all—between “believers” and “religiosity”. That is, in terms of identification, both terms imply an openness towards a form of faith or religiosity.

A notable strength in the present study relates to the analytic rigour with which our hypotheses were tested. We used logistic regression models as well as the more comprehensive medflex analysis and compared the output from both analyses to ensure the overall veracity of our statistical results. Furthermore, the populations-based approach, and the fact that the study population (recruited as part of the TOF pilot2 study) was not recruited to participate in a project focusing on religiosity and health, also represents a considerable strength. That is, it is unlikely that people chose not to participate in the TOF pilot2 study because they do not wish share information about their religious practice. Finally, to our knowledge, this is the first study to investigate whether social network mediates the association between religiosity and health behaviour among people with a more individualised and private religiosity.

## Future Research

In future studies, it would be interesting to include a focus on the stress-buffering effects of social connectedness, which is another potential pathway linking religiosity and health and health behaviour (Ozbay et al. 2008). For example, social connectedness may buffer against persistent psychological stressors and the associated physiological burden on the neuroendocrine system—a risk factor for a range of chronic illnesses (Schneiderman et al. 2005; Umberson & Karas Montez, 2010). Further, and of particular relevance to the present paper, the stress-buffering effects of social connectedness may also positively impact on the individual’s opportunity, self-efficacy, and motivation for healthy living. Specifically, studies have shown that psychological stress impacts negatively on health behaviour (Cheon et al., 2020). Thus, by facilitating resilience to stress, social connectedness may endow the individual with the mental energy and fortitude to deal with stress in a more considered and healthy manner.

Previous research has noted the importance of social norms as an explanatory factor in the relationship between religiosity and health (Ellison & Levin, 1998). Specifically, the descriptive (how do people behave) and injunctive (how should people behave) norms that define a particular group or network often guide individual behaviour. That is, if healthy social norms are perceived in a group with which the individual identifies (e.g. a church congregation), then this will often impact on individual choices and lifestyle. Accounting for perceived norms may have shed more light on the social network pathway that we examined in this study. Given the secondary nature of our data, however, we were not able to include a social norms measure. Future research into this particular mechanism as it relates to private vs. public religiosity and health would thus be valuable.

## Conclusion

To our knowledge, this study is the first to investigate whether social networks mediate the association between religiosity and health behaviour in a Danish sample. In contrast to past research, our analyses detected no such mediating effect. Our results did, however, indicate some health-behavioural benefits associated with religiosity over non-religiosity—particularly in terms of diet. We contextualise these results within the broader literature, noting several strengths and limitations in our data and analytic approach that hopefully will inform future research endeavours in this area. Ultimately, by distinguishing between private and public religiosity, this study represents an initial step towards uncovering the nature of the association between different variations of religiosity and health outcomes.

## Supplementary Information

Below is the link to the electronic supplementary material.

Supplementary file1 (DOCX 66 KB)

## References

[CR1] 5 facts about the religious lives of African Americans. (2018). Retrieved from https://www.pewresearch.org/fact-tank/2018/02/07/5-facts-about-the-religious-lives-of-african-americans/

[CR2] Ahrenfeldt LJ, Møller S, Andersen-Ranberg K, Vitved AR, Lindahl-Jacobsen R, Hvidt NC (2017). Religiousness and health in Europe. European Journal of Epidemiology.

[CR3] Americans are far more religious than adults in other wealthy nations. (2018). Retrieved from https://www.pewresearch.org/fact-tank/2018/07/31/americans-are-far-more-religious-than-adults-in-other-wealthy-nations/

[CR4] An adjusted preventive program against lifestyle related diseases (TOFpilot2). (2019). Retrieved from https://clinicaltrials.gov/ct2/show/NCT03913585?term=TOF&cntry=DK&draw=2&rank=5

[CR5] Anbefalinger om fysisk aktivitet for voksne under 65 år. (2019). Retrieved from https://www.sst.dk/da/viden/fysisk-aktivitet/anbefalinger-om-fysisk-aktivitet/voksne-under-65-aar

[CR6] Andersen PB, Lüchau P, Gundelach P (2011). Individualisering og aftraditionalisering af danskernes religiøse værdier. Små og store forandringer - Danskernes værdier siden 1981.

[CR7] Cheadle, J. E., & Schwadel, P. (2012). The ‘friendship dynamics of religion,’ or the ‘religious dynamics of friendship’? A social network analysis of adolescents who attend small schools. *Social Science Research, 41*(5), 1198–1212. 10.1016/j.ssresearch.2012.03.014 Retrieved from https://www.sciencedirect.com/science/article/pii/S0049089X1200066X. https://www.ncbi.nlm.nih.gov/pmc/articles/PMC3461188/pdf/nihms367881.pdf 10.1016/j.ssresearch.2012.03.014 PMC3461188 23017927

[CR8] Cheon, Y., Park, J., Jeong, B. Y., Park, E. Y., Oh, J. K., Yun, E. H., & Lim, M. K. (2020). Factors associated with psychological stress and distress among Korean adults: the results from Korea National Health and Nutrition Examination Survey. *Scientific Reports, 10*(1), 15134. 10.1038/s41598-020-71789-y Retrieved from https://www.ncbi.nlm.nih.gov/pmc/articles/PMC7492217/pdf/41598_2020_Article_71789.pdf 10.1038/s41598-020-71789-y PMC7492217 32934275

[CR9] Christensen, J. O., Sandbaek, A., Lauritzen, T., & Borch-Johnsen, K. (2004). Population-based stepwise screening for unrecognised Type 2 diabetes is ineffective in general practice despite reliable algorithms. *Diabetologia, 47*(9), 1566–1573. 10.1007/s00125-004-1496-2 Retrieved from https://link.springer.com/content/pdf/10.1007%2Fs00125-004-1496-2.pdf 10.1007/s00125-004-1496-2 15365615

[CR10] Debnam, K., Holt, C. L., Clark, E. M., Roth, D. L., & Southward, P. (2012). Relationship between religious social support and general social support with health behaviors in a national sample of African Americans. *Journal of Behavioral Medicine, 35*(2), 179–189. 10.1007/s10865-011-9338-4 Retrieved from https://pubmed.ncbi.nlm.nih.gov/21487724/10.1007/s10865-011-9338-4 PMC3336193 21487724

[CR11] Den danske værdiundersøgelse 1981–2017. (2019). Retrieved from https://www.sa.dk/da/forskning-rigsarkivet/benyt-surveydata/vaerdiundersoegelsen/

[CR12] Ellison, C. G., & Levin, J. S. (1998). The religion-health connection: evidence, theory, and future directions. *Health Education and Behavior, 25*(6), 700–720. 10.1177/109019819802500603 Retrieved from https://pubmed.ncbi.nlm.nih.gov/9813743/10.1177/109019819802500603 9813743

[CR13] Hall, D. E., Meador, K. G., & Koenig, H. G. (2008). Measuring Religiousness in Health Research: Review and Critique. *Journal of religion and health, 47*(2), 134–163. 10.1007/s10943-008-9165-2 Retrieved from https://pubmed.ncbi.nlm.nih.gov/19105008/10.1007/s10943-008-9165-2 PMC8823950 19105008

[CR14] Haslam SA, Jetten J, Postmes T, Haslam C (2009). Social Identity, Health and Well-Being: An Emerging Agenda for Applied Psychology. Applied Psychology: an International Review.

[CR15] Haslam, C., Holme, A., Haslam, S. A., Iyer, A., Jetten, J., & Williams, W. H. (2008). Maintaining group memberships: Social identity continuity predicts well-being after stroke. *Neuropsychological Rehabilitation, 18*(5–6), 671–691. 10.1080/09602010701643449 Retrieved from https://pubmed.ncbi.nlm.nih.gov/18924001/10.1080/09602010701643449 18924001

[CR16] Hastings, O. P. (2016). Not a lonely crowd? Social connectedness, religious service attendance, and the spiritual but not religious. *Social Science Research, 57*, 63–79. 10.1016/j.ssresearch.2016.01.006 Retrieved from https://www.sciencedirect.com/science/article/pii/S0049089X16000272. https://www.sciencedirect.com/science/article/pii/S0049089X16000272?via%3Dihub 10.1016/j.ssresearch.2016.01.006 26973032

[CR17] Holt CL, Wang MQ, Clark EM, Williams BR, Schulz E (2013). Religious involvement and physical and emotional functioning among African Americans: The mediating role of religious support. Psychology & Health.

[CR18] Holt, C. L., Roth, D. L., Huang, J., & Clark, E. M. (2018). Role of religious social support in longitudinal relationships between religiosity and health-related outcomes in African Americans. *Journal of Behavioral Medicine, 41*(1), 62–73. 10.1007/s10865-017-9877-4 Retrieved from https://pubmed.ncbi.nlm.nih.gov/28776192. https://www.ncbi.nlm.nih.gov/pmc/articles/PMC5766361/10.1007/s10865-017-9877-4 PMC5766361 28776192

[CR19] Hvidt, N. C., Hvidtjørn, D., Christensen, K., Nielsen, J. B., & Søndergaard, J. (2017). Faith Moves Mountains—Mountains Move Faith: Two Opposite Epidemiological Forces in Research on Religion and Health. *Journal of Religion and Health, 56*(1), 294–304. 10.1007/s10943-016-0300-1 Retrieved from https://www.ncbi.nlm.nih.gov/pmc/articles/PMC5222926/pdf/10943_2016_Article_300.pdf 10.1007/s10943-016-0300-1 PMC5222926 27541015

[CR20] Kim, K. H., & Sobal, J. (2004). Religion, social support, fat intake and physical activity. *Journal of Public Health and Nutrition 7*(6), 773–781. 10.1079/PHN2004601 Retrieved from https://www.cambridge.org/core/journals/public-health-nutrition/article/religion-social-support-fat-intake-and-physical-activity/97CBC1C3C36DE1C4B1FFB3881B4C2A54 10.1079/phn2004601 15369616

[CR21] Kobayashi, D., Shimbo, T., Takahashi, O., Davis, R. B., & Wee, C. C. (2015). The relationship between religiosity and cardiovascular risk factors in Japan: a large-scale cohort study. *Journal of the American Society of Hypertension, 9*(7), 553–562. 10.1016/j.jash.2015.04.003 Retrieved from https://pubmed.ncbi.nlm.nih.gov/26188400/10.1016/j.jash.2015.04.003 PMC4597307 26188400

[CR22] Koenig HG, King DE, Carson VB (2012). Handbook of Religion and Health.

[CR23] Larsen LB, Sonderlund AL, Sondergaard J, Thomsen JL, Halling A, Hvidt NC, Thilsing T (2018). Targeted prevention in primary care aimed at lifestyle-related diseases: a study protocol for a non-randomised pilot study. BMC Family Practice.

[CR24] Lim C, Putnam RD (2010). Religion, social networks, and life satisfaction. American Sociological Review.

[CR25] Lubben, J., Iliffe, S., Stuck, A. E., Beck, J. C., von Renteln Kruse, W., Blozik, E., & Gillmann, G. (2006). Performance of an Abbreviated Version of the Lubben Social Network Scale Among Three European Community-Dwelling Older Adult Populations. *The Gerontologist, 46*(4), 503–513. 10.1093/geront/46.4.503 Retrieved from https://academic.oup.com/gerontologist/article/46/4/503/623897 10.1093/geront/46.4.503 16921004

[CR26] Moreau, C., Trussell, J., & Bajos, N. (2013). Religiosity, religious affiliation, and patterns of sexual activity and contraceptive use in France. *European Journal of Contraception and Reproductive Health Care, 18*(3), 168–180. 10.3109/13625187.2013.777829 Retrieved from https://www.ncbi.nlm.nih.gov/pmc/articles/PMC3656140/pdf/nihms447847.pdf 10.3109/13625187.2013.777829 PMC3656140 23547890

[CR27] Nordfjærn T (2018). Religiosity and alcohol use: Is religiosity important for abstention and consumption levels in the second half of life?. Substance Use and Misuse.

[CR28] Oman D, Thoresen CE (2002). 'Does religion cause health?': Differing interpretations and diverse meanings. Journal of Health Psychology.

[CR29] Ozbay, F., Fitterling, H., Charney, D., & Southwick, S. (2008). Social support and resilience to stress across the life span: a neurobiologic framework. *Current psychiatry reports, 10*(4), 304. 10.1007/s11920-008-0049-7 Retrieved from https://pubmed.ncbi.nlm.nih.gov/18627668/10.1007/s11920-008-0049-7 18627668

[CR30] Reeves, R. R., Adams, C. E., Dubbert, P. M., Hickson, D. A., & Wyatt, S. B. (2012). Are religiosity and spirituality associated with obesity among African Americans in the Southeastern United States (the Jackson Heart Study)? *Journal of Religion and Health, 51*(1), 32–48. 10.1007/s10943-011-9552-y Retrieved from https://pubmed.ncbi.nlm.nih.gov/22065213/10.1007/s10943-011-9552-y PMC5324976 22065213

[CR31] Rosen I (2009). I’m a Believer – But I’ll Be Damned if I’m Religious : Belief and Religion in the Greater Copenhagen Area : A Focus Group Study.

[CR32] Schneiderman, N., Ironson, G., & Siegel, S. D. (2005). Stress and health: psychological, behavioral, and biological determinants. *Annual Review of Clinical Psychology, 1*, 607–628. 10.1146/annurev.clinpsy.1.102803.144141 Retrieved from https://www.ncbi.nlm.nih.gov/pmc/articles/PMC2568977/pdf/nihms70622.pdf 10.1146/annurev.clinpsy.1.102803.144141 PMC2568977 17716101

[CR33] Schnell T (2009). The Sources of Meaning and Meaning in Life Questionnaire (SoMe): Relations to demographics and well-being. The Journal of Positive Psychology.

[CR34] Schwadel P, Hardy SA (2022). What aspects of religiosity are associated with values?. Journal for the Scientific Study of Religion.

[CR35] Socialstyrelsen. (2011). *Nationella riktlinjer för sjukdomsförebyggande metoder 2011. Tobaksbruk, riskbruk av alkohol, otillräcklig fysisk aktivitet och ohälsosamma matvanor* (S. Socialstyrelsen Ed.). Västerås.

[CR36] Statistik om Digital Post. (2020). Retrieved from https://digst.dk/it-loesninger/digital-post/om-loesningen/tal-og-statistik-om-digital-post/

[CR37] Stort fald i antallet af gruppe 2-patienter. (2017). Retrieved from https://www.laeger.dk/nyhed/stort-fald-i-antallet-af-gruppe-2-patienter

[CR38] Svensson, N. H., Hvidt, N. C., Nissen, S. P., Storsveen, M. M., Hvidt, E. A., Søndergaard, J., & Thilsing, T. (2019). Religiosity and health-related risk behaviours in a secular culture-is there a correlation? *Journal of Religion and Health*. 10.1007/s10943-019-00919-2 Retrieved from https://www.ncbi.nlm.nih.gov/pmc/articles/PMC7502034/10.1007/s10943-019-00919-2 PMC7502034 31562592

[CR39] Thilsing, T., Larsen, L. B., Sonderlund, A. L., Andreassen, S. S., Christensen, J. R., Svensson, N. H., . . . Sondergaard, J. (2021). Effects of a Co-Design–Based Invitation Strategy on Participation in a Preventive Health Check Program: Randomized Controlled Trial. *JMIR Public Health and Surveillance, 7*(3), e25617. 10.2196/25617 Retrieved from https://publichealth.jmir.org/2021/3/e25617 10.2196/25617 PMC7991992 33688836

[CR40] U.S. adults are more religious than Western Europeans. (2018). Retrieved from https://www.pewresearch.org/fact-tank/2018/09/05/u-s-adults-are-more-religious-than-western-europeans/

[CR41] Umberson D, Karas Montez J (2010). Social relationships and health: A flashpoint for health policy. Journal of Health and Social Behavior.

[CR42] Vigliotti, V., Taggart, T., Walker, M., Kusmastuti, S., & Ransome, Y. (2020). Religion, faith, and spirituality influences on HIV prevention activities: A scoping review. *PloS One, 15*(6), e0234720. 10.1371/journal.pone.0234720 Retrieved from https://www.ncbi.nlm.nih.gov/pmc/articles/PMC7297313/pdf/pone.0234720.pdf 10.1371/journal.pone.0234720 PMC7297313 32544212

[CR43] Yeary, K. H.-c. K., Ounpraseuth, S., Moore, P., Bursac, Z., & Greene, P. (2012). Religion, Social Capital, and Health. *Review of Religious Research, 54*(3), 331–347. 10.1007/s13644-011-0048-8 Retrieved from http://www.jstor.org/stable/41940791

[CR44] Ysseldyk, R., Haslam, S. A., & Haslam, C. (2013). Abide with me: religious group identification among older adults promotes health and well-being by maintaining multiple group memberships. *Aging & Mental Health, 17*(7), 869–879. 10.1080/13607863.2013.799120 Retrieved from https://pubmed.ncbi.nlm.nih.gov/23711247/10.1080/13607863.2013.799120 23711247

[CR45] Zuckerman P (2008). Samfund uden Gud.

