# Supplementary material for: The Association Between Individualised Religiosity and Health Behaviour in Denmark: Are Social Networks a Mediating Factor?
Source: J Relig Health. 2022 Sep 9;61(6):4738–57. doi: 10.1007/s10943-022-01650-1 (PMC9569303; doi:10.1007/s10943-022-01650-1)
Supplement: Supplementary file 1 — Supplementary file1 (DOCX 66 KB) [file 10943_2022_1650_MOESM1_ESM.docx]

**Supplementary materials**

**Figure 1: Box plot of the social network sum score in the four religiosity categories**

**
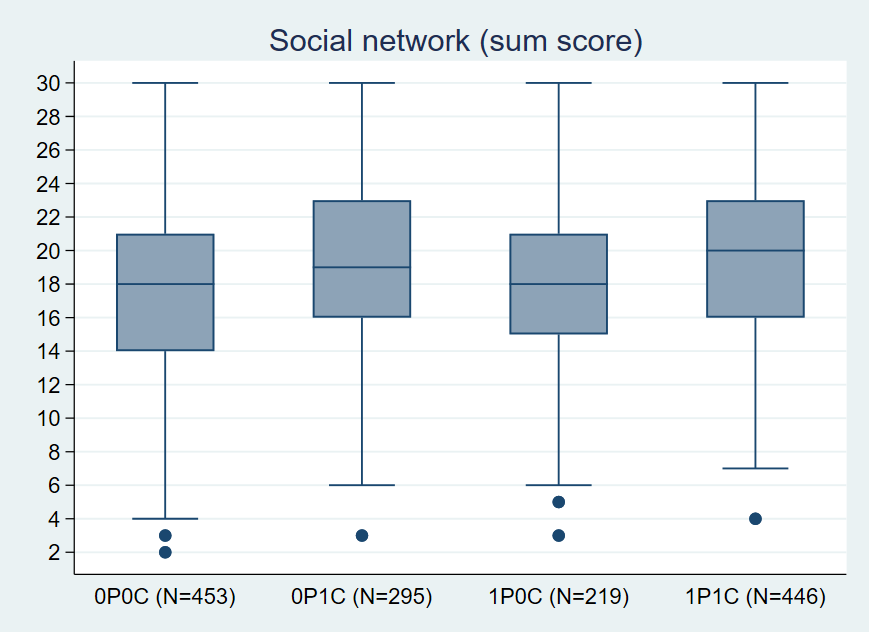
**

*****Note: No religiosity (0P0C): Closed towards both prayer/meditation practice and church/mosque attendance, public religiosity-church only (0P1C): Closed towards prayer/meditation practice but open towards church/mosque attendance, private religiosity-prayer only (1P0C): Open towards prayer/meditation practice bud closed towards church/mosque attendance, and public religiosity-church and prayer (1P1C): Open towards both prayer/meditation practice and church/mosque attendance.

**Supplementary materials**

**Table 1: Characteristic of the study populations religiosity by either strong or weak social network**

|  |  | Weak social network (score<12) | Strong social network (score≥12) | Total |
| --- | --- | --- | --- | --- |
|  | **Total [n (%)]** | **143 (100.0)** | **1270 (100.0)** | **1413 (100.0)** |
| Religiosity | No religiosity (0P0C) | 68 (47.5) | 385 (30.3) | 453 (32.0) |
|  | Public religiosity-church only (0P1C) | 21 (14.7) | 274 (21.6) | 295 (20.9) |
|  | Private religiosity-prayer only (1P0C) | 27 (18.9) | 192 (15.1) | 219 (15.5) |
|  | Public religiosity-church and prayer (1P1C) | 27 (18.9) | 419 (33.0) | 446 (31.6) |

*Note: No religiosity (0P0C): Closed towards both prayer/meditation practice and church/mosque attendance, public religiosity-church only (0P1C): Closed towards prayer/meditation practice but open towards church/mosque attendance, private religiosity-prayer only (1P0C): Open towards prayer/meditation practice bud closed towards church/mosque attendance, and public religiosity-church and prayer (1P1C): Open towards both prayer/meditation practice and church/mosque attendance.

**Table 2: Results from the logistics regression analyses**

|  | | | Healthy diet | | | Physical activity | | | | |
| --- | --- | --- | --- | --- | --- | --- | --- | --- | --- | --- |
|  |  | Model 1 | | | Model 2 | | Model 1 | | Model 2 | |
|  |  | **Unadjusted**  **OR (95% CI)** | | **Adjusted**  **OR (95% CI)** | **Unadjusted**  **OR (95% CI)** | **Adjusted**  **OR (95% CI)** | **Unadjusted**  **OR (95% CI)** | **Adjusted**  **OR (95% CI)** | **Unadjusted**  **OR (95%CI)** | **Adjusted**  **OR (95% CI)** |
| No religiosity (0P0C) |  | ref. | | ref. | ref. | ref. | ref. | ref. | ref. | ref. |
| Public religiosity-church only (0P1C) |  | 1.92 (1.36;2.70) | | 1.68 (1.18;2.39) | 1.81 (1.28;2.56) | 1.61 (1.13;2.30) | 1.76 (1.11;2.81) | 1.65 (1.02;2.65) | 1.61 (1.01;2.58) | 1.54 (0.96;2.48) |
| Private religiosity-prayer only (1P0C) |  | 1.27 (0.89;1.80) | | 1.07 (0.74;1.54) | 1.25 (0.88;1.78) | 1.04 (0.72;1.51) | 0.93 (0.60;1.44) | 0.89 (0.56;1.39) | 0.91 (0.59;1.41) | 0.86 (0.55;1.36) |
| Public religiosity-church and prayer (1P1C) |  | 2.56 (1.86;3.51) | | 2.08 (1.49;2.90) | 2.39 (1.74;3.30) | 1.96 (1.40;2.74) | 1.52 (1.03;2.26) | 1.45 (0.96;2.20) | 1.37 (0.92;2.04) | 1.32 (0.87;2.01) |
| Social network | Strong (score≥12) | 1.61 (1.11;2.34) | | 1.46 (0.99;2.14) |  |  | 1.56 (0.99;2.45) | 1.47 (0.91;2.35) |  |  |
|  | Weak (score<12) | ref. | | ref. |  |  | ref. | ref. |  |  |
|  | Sum score |  | |  | 1.05 (1.03;1.08) | 1.05 (1.02;1.07) |  |  | 1.07 (1.04;1.10) | 1.07 (1.03;1.10) |
| Sex | Men |  | | 0.58 (0.45;0.76) |  | 0.58 (0.44;0.76) |  | 0.83 (0.59;1.17) |  | 0.83 (0.59;1.17) |
|  | Women |  | | ref. |  | ref. |  | ref. |  | ref. |
| Age group | 29-30 years |  | | ref. |  | ref. |  | ref. |  | ref. |
|  | 40-49 years |  | | 1.02 (0.72;1.45) |  | 1.01 (0.71;1.44) |  | 1.23 (0.81;1.88) |  | 1.23 (0.81;1.88) |
|  | 50-60 years |  | | 1.51 (1.06;2.16) |  | 1.51 (1.06;2.15) |  | 1.71 (1.11;2.62) |  | 1.72 (1.12;2.64) |
| Country of origin | Denmark |  | | 0.60 (0.27;1.36) |  | 0.54 (0.24;1.21) |  | 2.28 (1.13;4.63) |  | 1.89 (0.92;3.89) |
|  | Not Denmark |  | | ref. |  | ref. |  | ref. |  | ref. |
| Level of education | ≤10 years |  | | ref. |  | ref. |  | ref. |  | ref. |
|  | 10-15 years |  | | 1.07 (0.75;1.54) |  | 1.08 (0.75;1.54) |  | 1.25 (0.80;1.97) |  | 1.26 (0.80;1.98) |
|  | >15 years |  | | 1.84 (1.23;2.75) |  | 1.80 (1.20;2.69) |  | 1.22 (0.75;1.99) |  | 1.17 (0.72;1.91) |
| Cohabitation status | Cohabiting |  | | 1.37 (1.02;1.83) |  | 1.30 (0.97;1.75) |  | 0.77 (0.52;1.16) |  | 0.72 (0.48;1.08) |
|  | Single |  | | ref. |  | ref. |  | ref. |  | ref. |
| Employment status | Employee/self-employed |  | | 1.11 (0.76;1.63) |  | 1.07 (0.73;1.57) |  | 1.49 (0.96;2.33) |  | 1.37 (0.87;2.15) |
|  | Not employed |  | | ref. |  | ref. |  | ref. |  | ref. |

*Model 1 refers to the model including the binary social network variable whereas model 2 refers to the model including the continuous social network sum score variable.

**Adjusted for sex, age group, level of education, cohabitation status, country of origin, and employment status.

***Note: No religiosity (0P0C): Closed towards both prayer/meditation practice and church/mosque attendance, public religiosity-church only (0P1C): Closed towards prayer/meditation practice but open towards church/mosque attendance, private religiosity-prayer only (1P0C): Open towards prayer/meditation practice bud closed towards church/mosque attendance, and public religiosity-church and prayer (1P1C): Open towards both prayer/meditation practice and church/mosque attendance.
